# A Metagenomic and Gene Expression Analysis in Wheat (*T. durum*) and Maize (*Z. mays*) Biofertilized with PGPM and Biochar

**DOI:** 10.3390/ijms231810376

**Published:** 2022-09-08

**Authors:** Sara Graziano, Marina Caldara, Mariolina Gullì, Annamaria Bevivino, Elena Maestri, Nelson Marmiroli

**Affiliations:** 1 Interdepartmental Center SITEIA.PARMA, University of Parma, Parco Area delle Scienze, 43124 Parma, Italy; 2 Department of Chemistry, Life Sciences and Environmental Sustainability, University of Parma, Parco Area delle Scienze 11/A, 43124 Parma, Italy; 3 Department for Sustainability, Italian National Agency for New Technologies, Energy and Sustainable Economic Development, ENEA Casaccia Research Center, 00123 Rome, Italy; 4 National Interuniversity Consortium for Environmental Sciences (CINSA), 30123 Venice, Italy

**Keywords:** biofertilizer, biochar, *Zea mays*, *Triticum durum*, gene expression, rhizospheric microbes, soil pollution

## Abstract

Commodity crops, such as wheat and maize, are extremely dependent on chemical fertilizers, a practice contributing greatly to the increase in the contaminants in soil and water. Promising solutions are biofertilizers, i.e., microbial biostimulants that when supplemented with soil stimulate plant growth and production. Moreover, the biofertilizers can be fortified when (i) provided as multifunctional consortia and (ii) combined with biochar with a high cargo capacity. The aim of this work was to determine the molecular effects on the soil microbiome of different biofertilizers and delivery systems, highlight their physiological effects and merge the data with statistical analyses. The measurements of the physiological parameters (i.e., shoot and root biomass), transcriptomic response of genes involved in essential pathways, and characterization of the rhizosphere population were analyzed. The results demonstrated that wheat and maize supplemented with different combinations of selected microbial consortia and biochar have a positive effect on plant growth in terms of shoot and root biomass; the treatments also had a beneficial influence on the biodiversity of the indigenous rhizo-microbial community, reinforcing the connection between microbes and plants without further spreading contaminants. There was also evidence at the transcriptional level of crosstalk between microbiota and plants.

## 1. Introduction

An increasing world population is challenging current agricultural production to ensure a steady food supply, a problem that is worsened by the striking losses of arable land and crop yields [1,2]. Wheat (*T. durum* and *T. aestivum*), the most important staple crop in the world, and maize (*Zea mays* L.) contribute to ~12.4% of the world’s food demand (3% of all cereals) and rank first in production volume worldwide (1.135 million tons) [3]. Their cultivation has a strong impact on the use of chemical fertilizers; in 2019, 26 and 27.26 Gt of fertilizers were used for wheat and maize, respectively (https://www.statista.com/ accessed on 12 June 2022). In the future, enhancing their cultivation will be a global challenge [4] that requires the implementation of “sustainable agriculture” strategies, which have been strengthened over time by both the UN (United Nations) and FAO (Food and Agriculture Organization) and reiterated within the 17 Sustainable Development Goals of the 2030 Agenda.

One of the main challenges is managing plant fertilization since chemical fertilizers, which are used at a high rate, contribute to both inorganic (metals, metalloids, and radionuclides) and organic contaminants (dichlorodiphenyltrichloroethane, polychlorinated biphenyls, phthalates, dioxin). Organic fertilizers, on the other hand, are not completely void of contaminants. Animal manure and sludge from municipal water treatment plants can bring benefits to the soil in terms of organic matter but they are also a source of organic and inorganic contaminants [5,6]. The production of inorganic fertilizer is energy intensive; it has been estimated that 2% of the world’s energy production is devoted to the production of inorganic nitrogen fertilizers, generating 465 million tons of CO_2_ [7]. Nanofertilizers have several advantages: lower persistence in the soil, slower release, precision delivery of active compounds, higher efficacy at the level of the targets, and the possibility of use in biofortification [8]. They have also some drawbacks such as costs and subsidies, application mode, heterogeneity of distribution, scaling up of technologies, legislative framework, and acceptance by farmers [8,9] (Figure S1).

Biotechnologies can contribute to the development of useful practices for sustainable agriculture [10], for example, the use of biostimulants, which have been defined as products able to stimulate natural processes, when applied to plants or soil, increase the absorption of nutrients, tolerance to biotic and abiotic stresses, and crop quality [3]. The types of biostimulants are biofertilizers, bacteria, and/or fungi, defined as plant growth-promoting microbes (PGPM), which establish a positive relationship with the plant by increasing the bioavailability of many nutrients present in the soil and have a positive impact on plant yield and health [6,11,12]. To improve the performance of biofertilizers, it is possible to combine them with soil amendments, components that can positively change some parameters of soil fertility (such as pH, organic material content, cation exchange capacity, nutrient retention capacities) and can stimulate microbial growth and survival [3,13,14]. Biochar, an amendment obtained by the pyrolysis or pyrogasification of renewable resources, sometimes considered waste, is a good candidate [15,16]. Moreover, its structural porosity makes it ideal to provide a niche in which microorganisms can survive environmental stress [14,17,18,19,20]. The use of biochar as a carrier of biofertilizers has been suggested [16,21] but to evaluate its feasibility, it is important to study the biochar effect on soil and plants in controlled growth conditions.

Moreover, the agricultural sector is strongly interested in making the cultivation of commodities such as maize and wheat more sustainable because of their relevance to human and animal nutrition, but this will need important changes in fertilization and energy consumption to avoid the production of pollutants [22,23]. Previous studies have evaluated the field application of mycorrhizae to maize [24] and wheat [25,26,27], obtaining promising results, as their application increased the root absorption of micronutrients from the soil [28,29,30,31]. Other studies have shown that the application of biofertilizers to wheat cultivation is hampered by the variability in plant responses and environmental conditions [32,33]. Therefore, any effective use of biostimulants in agriculture requires a better understanding of (i) the interactions between plants and biostimulants, (ii) the influence that these products have on the rhizosphere microbial communities, and (iii) the factors that have the most influence on the crosstalk between plants and microbes to promote only the beneficial ones.

This study investigated the effect of the combination of biochar (as a carrier), microbial consortia, and/or arbuscular mycorrhizal fungi (AMF) on durum wheat and maize when grown in greenhouses. The study involved (i) the evaluation of the effects of the different treatments on plant growth and physiology; (ii) the 16S rDNA and ITS profiling of the soil microbial communities to evaluate the main taxa and changes in the relative composition; (iii) a systematic PCA analysis of all data obtained; and (iv) merging the molecular and physiological data to produce a viable scenario for new plant fertilization. The study of the systemic changes induced in leaf tissues through the gene expression analysis of the presence of biochar and/or microorganisms demonstrated the plant and soil crosstalk in reacting to different environmental conditions (such as soil treatment) and plant age. A systematic biological approach showed some of the plant molecules involved.

## 2. Results and Discussion

### 2.1. Char Has a High Cargo Capacity for Microbes

The use of char in agriculture and its positive effects have been extensively reviewed [16,34,35,36,37]. Moreover, its porous structure represents a perfect habitat for microbial growth [17,38,39]. The high cargo capacity of char was utilized and demonstrated in this work. The microbial consortia chosen for the experiments were the MC-B and MC-C [40,41], previously tested on wheat *T. aestivum* [41] but not on wheat *T. durum* nor *Z. mays*. First, it was evaluated whether the microbes could colonize the char surface and functionalize it and the extent to which this was achieved. The results presented in Figure S2A show that each gram of char was colonized on average by 10^8^ to 10^10^ microbial cells, which were visible with the Syto-9 staining of char (Figure S2B,C). This is important information and shows that char at a working concentration was not toxic to the microbes and allowed a high cargo capacity (absence of biochar phytotoxicity was previously tested up to a concentration of 5% *w*/*v*; see biochar A4 in [16]). Alginate microbial encapsulation, or the stabilization of the microbes on inert materials (i.e., zeolite, bentonite, perlite, talc, or vermiculite), can also be employed to deliver microbes (see Table S1) [40,42]. The method most commonly used to deliver biofertilizers is the coating of individual seeds with a gluing chemical (i.e., methylcellulose) that was previously mixed with the desired microbes [43,44]. It was estimated that on average, the viable number of cells present on the coated seeds varied between 10^4^ to 10^8^ CFU/seed [45,46,47]. The influences exerted by PGPMs should be more effective when higher counts of viable microbes are delivered near the roots. Considering the average weight of maize and wheat seeds (respectively, 0.3 and 0.05 g) and the CFU/ seedthat can be reached by seed coating, functionalized char allows for the delivery of a higher number of microbial cells, on average in the range of 10 to 10^4^ more CFU g^−1^, as previously reported [47,48]. This, of course, does not exclude that seed coatings can be improved.

### 2.2. Effects of PGPM and Char on Plant Growth

The aims of this study were to compare the effects of the microbial delivery systems and consortia (MC-B and MC-C) on plant growth. The measurements taken for plants, as described in the Materials and Methods section, are reported in Table 1 for durum wheat and Table 2 for maize; in particular, measurements of the length, fresh weight (FW), dry weight (DW), and percentage of dry biomass of both roots and shoots, as well as the SPAD index, were taken at 60 days after sowing (DAS).

#### 2.2.1. Wheat

**Figure 1:**
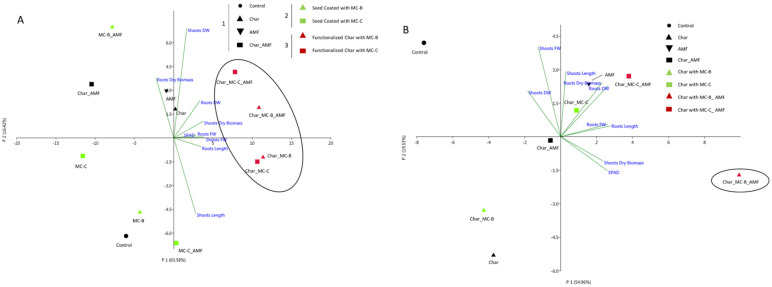
PCA analysis of the physiological growth data obtained for wheat (**A**) and maize (**B**) in response to different treatments. (**A**) Wheat treatments were 1 “Control” group (Control, Char, AMF, Char_AMF); 2 “Seed coating” group (MC-B, MC-C, MC-B_AMF, MC-C_AMF); 3 “Functionalized Char” group (Char_MC-B, Char_MC-C, Char_MC-B_AMF, Char_MC-C_AMF). (**B**) Maize treatments were “Control” group (Control, Char, AMF, Char_AMF); functionalized char group without AMF (Char_MC-B, Char_MC-C), and functionalized char with AMF (Char_MC-B_AMF, Char_MC-C_AMF). The colors and symbols are ordered according to the legend reported. The main groupings are indicated in circles.

The first comparison was of the microorganisms’ delivery systems, either seed coating or char. The different conditions used for the wheat experiment were grouped into three categories: 1 “Control”, 2 “Seed coating”, and 3 “ Functionalized Char”. The “Control” group included control, char (0.1% *w*/*w*), AMF (1.4% *w*/*w*), and Char_AMF. The “Seed coating” group included MC-B, MC-C, MC-B_AMF, and MC-C_AMF. The “Functionalized Char” group included: Char_MC-B, Char_MC-C, Char_MC-B_AMF, and Char_MC-C_AMF. The principal component analysis (PCA) (Figure 1A) showed that group 3 “Functionalized char” clustered along the first principal component axis (P1), whereas treatments 1 “Control” and 2 “Seed Coating” were widely distributed along the P2 axis.

All traits analyzed (see Table 1), except for shoot dry biomass, were the main factors in the P1 axis, accounting for 63.58% of the total variation. The shoot dry biomass was the main factor in the second P2 axis, accounting for 16.42% of the total variation.

Statistical analysis of shoot-related traits, such as shoot length, FW, DW, and the SPAD index, showed that the treatments of group 3 “Functionalized Char” with either MC-B or MC-C and AMF had significantly increased (*p* < 0.05) values with respect to both the group 1 “Control” and group 2 “Seed coating” treatments (Table 1). Char when used as a cargo system for MC-B and MC-C positively influenced these parameters. The group 3 treatments, Char_MC-B_AMF and Char_MC-C_AMF, showed significant differences (*p* < 0.05) for shoots FW and DW, and Char_MC-C_AMF also showed a significant difference for root length. For root and shoot dry biomass, the highest values were observed in the treatments for group 3 “Functionalized Char”, which were significantly different from both the control and seed-coating groups (*p* < 0.05). The values obtained guided the selection of the samples for the subsequent analysis of the 16S and ITS sequencing of microbiota from rhizospheric soil and for the gene expression analysis in leaf tissues. The eight wheat treatments selected were Control, Char, AMF, Char_AMF, MC-C, MC-C_AMF, Char_MC-C, and Char_MC-C_AMF.

#### 2.2.2. Maize

Considering the wheat results, the maize experiments were specifically directed to char as a delivery system. For this crop, the conditions tested were control, char, AMF, Char_AMF, Char_MC-B, Char_MC-B_AMF, Char_MC-C, and Char_MC-C_AMF.

The data collected were analyzed and the results are shown in Table 2.

No significant differences emerged from the data regarding the root FW, DW, and dry biomass. Instead, root length was the only trait that showed significant differences in the AMF, Char_MC-C_AMF, and Char-MC-B_AMF treatments, where the plants had longer roots than the control (*p* < 0.05). PCA analysis (Figure 1B) showed that the shoot and root length and dry biomass, root DW, and shoot chlorophyll content measured with SPAD were the main factors along the P1 axis, accounting for 54.96% of the total variation, whereas the shoot FW and DW were the main factors along P2 axis, accounting for 19.53% of the total variation. In particular, the treatment Char_MC-B_AMF determined a significant (*p* < 0.05) positive effect with respect to root length, shoot FW, DW, SPAD index, and dry biomass (Table 2). These results suggest a synergistic effect between the microorganisms and char, which led to a higher biomass and higher photosynthetic activity compared to the other treatments. Indeed, the analysis of the rhizospheric soil also showed that these treatments (with microbes and char) were influencing the ecology of the soil microbiome (see below).

The application of biofertilizers directly to seeds promotes plant growth from an early stage. The commercial requirements for an alternative to seed coating as a competitive delivery system demand a tool that also meets the high safety standards [49]. Studies including greenhouse and field trials have been focused on this topic for the last 20 years [50]. Quantitative data previously reported for wheat showed that char functionalization was more efficient than seed coating in boosting the biomass of both roots and shoots. Indeed, seed coating with carriers is a complex process requiring suitable moisture, temperatures, and nutrient availability to keep the bacteria alive over a sufficient period of time [51]. Instead, the physiological parameters considered suggested that char ensures optimal conditions for colonization by microorganisms as shown at the structural level (Figure S2). The functionalization of char with biofertilizers has been recently applied to other plants [44,52,53]. Moreover, the results obtained may be influenced by the properties of the microorganisms in the consortia such as the biological nitrogen fixation, synthesis of phytohormones (IAA, GA3, and cytokinin), and increased availability of micro- and macronutrients (phosphorus and iron) [54]. All data reported were consistent with the literature [2,31,53,55,56,57], that is, the presence of MC in combination with AMF determined an increase in root development, nutrient uptake, and root and shoot biomass. The novelty here is that the results were obtained using a new technology of functionalization and delivery with biochar and in two “in lab”-designed microbial consortia [40], which were not tested previously on wheat (*T. durum*) and maize (*Z. mays*).

### 2.3. Analysis of Rhizosphere Microbiota and Mycobiota

The results guided the selection of the wheat samples in which the rhizosphere bacterial and fungal communities were studied. Specifically, the samples chosen were those that displayed a stronger positive response to the presence of the biostimulants than their relative controls. Instead, all maize samples were considered.

#### 2.3.1. Bacteria and Fungi in the Rhizosphere of Wheat

**Figure 2:**
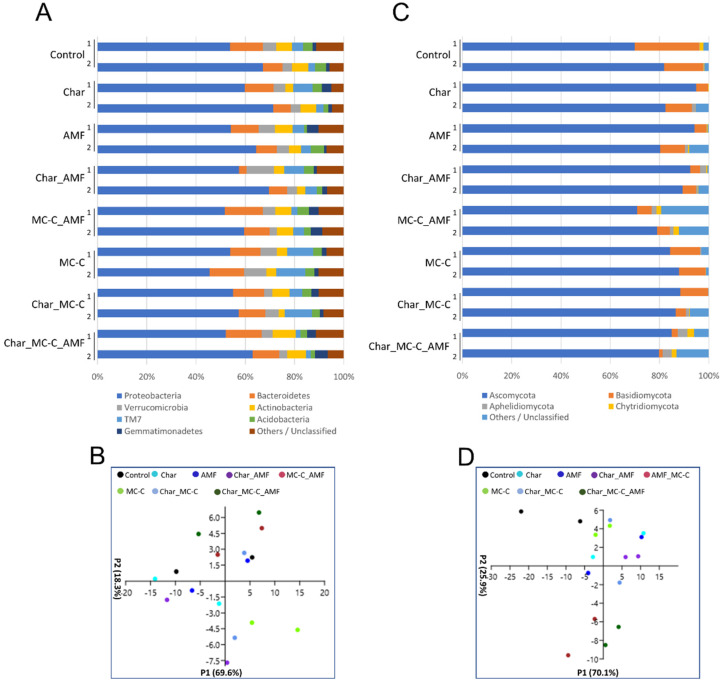
Phyla of rhizospheric bacterial and fungal communities in wheat soils untreated or treated with different combinations of biofertilizers. (**A**) Relative abundance (based on OTUs) of the most relevant bacterial phyla found in eight rhizospheric soils of wheat analyzed in duplicate (1–2); the colors are ordered from left to right according to the legend reported at the bottom of the panel; (**B**) PCA analysis of the eight selected bacterial communities in the different wheat soils analyzed. (**C**) Relative abundance (based on OTUs) of the most relevant fungal phyla found in eight rhizospheric soils of wheat analyzed in duplicate (1–2); the colors are ordered from left to right according to the legend reported at the bottom of the panel. (**D**) PCA analysis of eight selected fungal communities in the different wheat samples analyzed. Each symbol (circle) represents one replicate and the colors follow the legend reported at the top of the panel.

The most abundant bacterial phyla in all samples were Proteobacteria, accounting for over 55% of the total sequences, followed by Bacteroidetes with ~11% and then Verrucomicrobia, Actinobacteria, Acidobacteria, TM7 (Saccharibacteria), and Gemmatimonadetes, whose abundance ranged from 3 to 8% (Figure 2A). PCA was employed to generate a global overview of the data (Figure 2C). The Proteobacteria population was the main factor in the first principal component axis (P1), accounting for 69.6% of the total variation, whereas TM7 was the main factor in the second P2 axis. Proteobacteria include important species such as *Pseudomonas* sp. and *Burkholderia* sp. [58,59,60] and *Azospirillum* sp. and *Azotobacter* sp., which are nitrogen-fixing bacteria, whereas TM7 are hydrocarbon decomposers; however, so far, little is known about their characteristics as they have not yet been successfully isolated and cultivated [61,62]. Figure 2C,D show that the MC-C_AMF and Char_MC-C_AMF data were more similar. These results also show a similar combined effect of MC-C and AMF on the rhizospheric soil population, independent of their delivery method (see Materials and Methods Section 3.2 and Section 3.3). For Proteobacteria, the most represented classes were Alphaproteobacteria, Gammaproteobacteria, Saprospirae, Actinobacteria, and Betaproteobacteria, together accounting for over 60% of the retrieved sequences (Figure S3A).

Starting with the operational taxonomic units (OTUs), the Shannon diversity index and the estimator of the richness of Chao-1 were calculated (Table S2). The Shannon index increased in each of the treatments where the MCs were added and was at a minimum when only char was supplied. The differences were not particularly relevant but were expected. Indeed, char addition has been reported to modify the soil microbial community although its variable effects on soil depend on the soil type, char application rate, and char particle size [63,64,65]. It was expected that the addition of char to soil would positively influence the fungal population as fungi can better use the residues of the lignin still present in the char although this effect was not always observed [65,66]. Here, the rhizosphere fungal diversity was very low (Shannon indexes are all below 3) and a smaller Shannon index was measured when the soil was supplemented only with char, whereas the highest index was measured in the Char_MC-C_AMF treatment (Table S2). The main phyla present in all soils were Ascomycota, Basidiomycota, Aphelidiomycota, and Chytridiomycota (Figure 2B), representing, on average, more than 90% of the retrieved sequences. PCA analysis showed that the grouping of the samples MC-C_AMF and Char_ MC-C_AMF were separated from the others along the P2 axis and their variance was driven by changes in Ascomycota and Basidiomycota (Figure 2D). Indeed, in these two treatments, the relative abundance of Ascomycota and Basidiomycota decreased, a change that was accompanied by a parallel increase in other fungi phyla still uncharacterized; an effect that was reported to be stronger for the MC-C_AMF treatment.

#### 2.3.2. Bacteria and Fungi in the Rhizosphere of Maize

**Figure 3:**
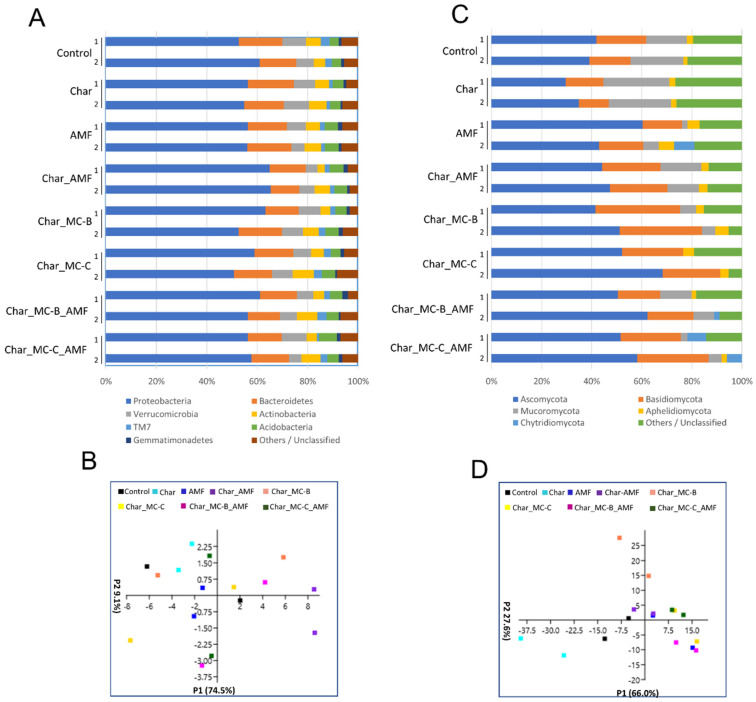
Phyla of rhizospheric bacterial and fungal communities in maize soils untreated or treated with different combinations of biofertilizers. (**A**) Relative abundance (based on OTUs) of the most relevant bacterial phyla found in eight rhizospheric soils of maize analyzed in duplicate (1–2); the colors are ordered from left to right according to the legend reported at the bottom of the panel; (**B**) PCA analysis of the eight selected bacterial communities in the different maize soils analyzed. (**C**) Relative abundance (based on OTUs) of the most relevant fungal phyla found in eight rhizospheric soil of maize analyzed in duplicate (1–2); the colors are ordered from left to right according to the legend reported at the bottom of the panel (**D**). PCA analysis of eight fungal communities in the different maize samples analyzed. Each symbol (squares) represents one replicate and the colors follow the legend reported at the top of the panel.

The most represented phyla retrieved in the rhizosphere soil of maize resembled those found for wheat (Figure 3A) and their relative abundance was also comparable. For the bacterial population, PCA analysis did not show any groupings (Figure 3C) and the Shannon and Chao-1 indices were all similar among the treatments (Table S2). The principal component P1 explained 74.5% of the observed variation (Figure 3C), an effect correlated mainly to Proteobacteria, whereas P2 accounted for 9.1% of the variation, an effect mostly correlated to Verrucomicrobia and Actinobacteria. Verrucomicrobia are phylogenetically heterogeneous Gram-negative bacteria, often described as inactive [67].

Actinobacteria have an important ecological role because they can degrade different types of contaminants (i.e., pesticides, herbicides, and fungicides). The non-Streptomyces *Arthrobacter* and *Rhodococccus* are among the most well-known genera [68,69,70,71]. The fungal population displayed evident differences between wheat and maize. In maize, the main fungal phyla were Ascomycota, Basidiomycota, Aphelidiomycota, and Chytridiomycota, followed by Mucoromycota, which accounted for ~5% in the control and char samples but dropped below 1% in all the other treatments (Figure 3B). Mucoromycota members have been reported either as beneficial or as pathogenic depending on the order [72]. The Mucoromycota present in the maize soil belong to the order of Mucorales, which have been annotated as opportunistic pathogens. It is, therefore, interesting that all the microbial treatments lowered the concentration of these fungi, with potential benefits to the overall health status of the soil. This effect was registered by the Shannon index, which was the highest in the control and char samples but was diminished in all the other treatments, an effect connected to a decrease in the Mucoromycota population. The PCA analysis showed that most of the data were grouped in a central cloud, whereas the values for the control and char samples were more separated (Figure 3D). The latter was distributed mainly along the P1 axis (P1 66% of Figure 3D) and their variability, as expected, depended mainly on the Ascomycota and Mucoromycota populations. The classes mostly represented within the fungal population were Sordariomycetes, Eurotiomycetes, Orbiliomycetes, Saccharomycetes, Mucororomycetes, and Agaromycetes (Figure S4). Mucoromycetes and Orbiliomycetes were basically absent in the wheat rhizosphere soil. Mucoromycetes belong to the phyla of Mucoroycota and they have been discussed above; Orbiliomycetes belong to the Ascomycota phylum and are involved in the biological control of nematodes [73]. Within the analyzed samples, Orbiliomycetes were particularly abundant in maize soil treated with AMF where they reached over 20% of abundance. Overall, fungi displayed the largest variability with the different treatments, which agrees with the results reported in other works [74,75,76].

### 2.4. Gene Expression in Wheat and Maize Leaves (at Two Different Growth Stages)

The variations observed in the microbial population at the level of the rhizosphere in wheat and maize offered the possibility of testing whether these treatments also determined a new type of crosstalk between the microorganisms and plants in the soil.

This was analyzed at the level of gene expression measuring the transcriptional variations that occur in genes involved in pathways essential for the plant. The selection of these target genes also considered previous data on transcriptional profiling in maize and wheat grown in a greenhouse and treated with biostimulants [4,77,78]. The rationale for the selection reflected the following criteria: (i) gene expression specifically in leaves; (ii) differential expression in response to biostimulants; and (iii) functions related to plant essential pathways.

With these criteria, 14 genes were chosen all highly modulated in response to various biostimulants in maize and wheat [4,77]. Our approach also included an interatomic analysis to evaluate the robustness of the chosen genes. The network of target genes was composed of 53 nodes and 275 edges and it had a highly significant enrichment *p*-value (<1.0 × 10^−16^). The average node degree, which is the average number of edges connecting all the nodes in the network of each node, was 10.4 supporting the robustness of the chosen genes (Figure S5).

The biological functions of the selected genes were photosynthesis, lipid metabolism, glycolysis and gluconeogenesis, starch biosynthesis, amino acid metabolism, and secondary metabolite biosynthesis (Table S3).

Wheat in greenhouse conditions is generally more responsive to biostimulants in the early stages, tillering, and stem elongation, than in the later stages. After 60 days from the inoculation of PGPM, the maximum increase in the shoot was up to +23% [53]. Therefore, the gene expression analysis of leaves during the early stages of growth at 21 DAS and at the end of the experiment at 60 DAS, was considered a good system for studying whether these effects were also accompanied by molecular crosstalk.

#### 2.4.1. Wheat

**Figure 4:**
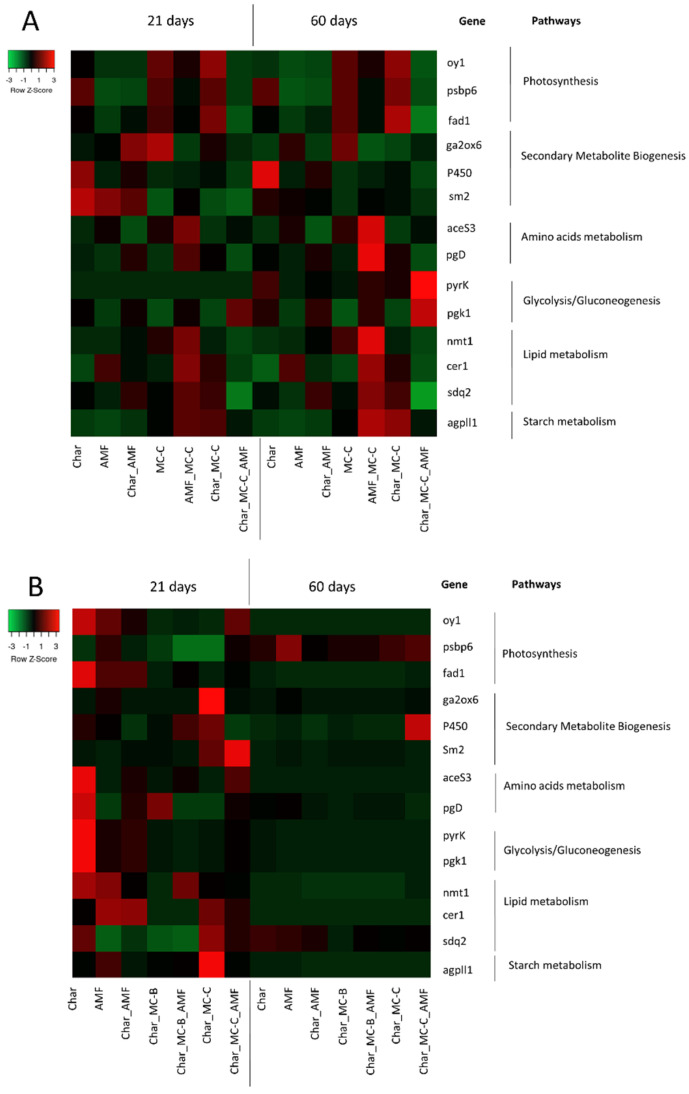
Heatmap showing the fold-change values in the 14 target genes analyzed in leaves at 21 and 60 DAS in response to the eight different treatments in wheat (**A**) and maize (**B**). For each gene, the corresponding pathway is indicated. Each row represents a gene and each column represents a treatment. Red and green correspond, respectively, to low and high expression levels. All values were normalized with respect to the control, which is therefore not shown.

As shown in Figure 4A, all 14 genes were thought to display a modulation in response to the different treatments and as a function of the developmental stages at 21 and 60 DAS. Moreover, the modulation by the various treatments was different from the control conditions.

At 21 DAS, the genes that were upregulated in response to treatment with a microbial consortium fell within four metabolic pathways: amino acid metabolism (*pgd*), glycolysis/gluconeogenesis (*pgk* and *pyrk*), secondary metabolite biogenesis (*P450*), and lipid metabolism (*nmt1*). Rhizosphere microbiotas seemed to play a key role in this context, with the plant roots determining the crosstalk that extended its effects at the level of plant growth and fitness, including the molecular effects at the levels of the genes involved in the essential pathways [79].

At 60 DAS, similar sets of genes were modulated in response to the same treatments compared to the control conditions. At this stage of growth, the treatment with a microbial consortium determined the induction of the genes involved in the four metabolic pathways essential for survival: lipid and starch metabolism (*cer1*, *nmt1*, *sdq2*, *agpll1*), photosynthesis (*oy1*, *psbp6*, *fad1*), amino acid metabolism (*aceS3*, *pgd*), and glycolysis/gluconeogenesis (*pyrk* and *pgk*).

PCA analysis was performed considering all plant traits for biomass and gene expression data (Figure S6). The information obtained through this approach underlined that the treatments with MC-C used in combination with char, AMF, or both, were distributed along the first principal component axis (PC 1) (29.44%) and were separated from all the other treatments. Therefore, the use of functionalized char determined a different growth condition with respect to the control conditions and the conditions of the functionalized seeds.

#### 2.4.2. Maize

As shown in Figure 4B, all 14 genes were modulated in response to the different treatments and as a function of the developmental stage at 21 and 60 DAS. Moreover, the modulation by the various treatments was different from the control conditions.

At 21 DAS, the genes that were upregulated belonged to six metabolic pathways: amino acid metabolism (*pgd*), starch metabolism (*agpll1*), photosynthesis (*fad1*, *oy1*), lipid metabolism (*cer1*, *nmt1*), secondary metabolite biogenesis (*P450*, *sm2*), and glycolysis/gluconeogenesis (*pgk*, *pyrk*). Plants treated with MC-B or C showed that the genes involved in the photosynthesis pathway were strongly up-regulated compared to the control conditions. The condition Char_ MC-C_ AMF determined a strong induction (FC > 4) of 9 genes (*aceS3*, *agpll1*, *cer1*, *fad1*, *nmt1*, *oy1*, *pgk,* and *pyrk*) involved in different metabolic pathways.

At 60 DAS, most of the target genes were downregulated (Figure 4B). The treatment Char_MC-C_AMF determined the induction of the genes involved in the lipid metabolism, photosynthesis, glycolysis/gluconeogenesis, and biogenesis of secondary metabolites. These data suggest a bioprotective effect of the microbial consortia when combined with AMF and char [80].

PCA analysis (Figure S7) correlated to the plant biomass and transcriptional data; the two growth conditions of char functionalized with both MC and AMF were grouped along the first principal components axis PC1 (21.30%) with respect to the other conditions analyzed. Therefore, the use of functionalized char had a distinct growth and gene expression effect compared to the control.

### 2.5. Correlation between Physiological and Molecular Data

The combination of char and microbial consortia was suggested in several instances and with different argumentations, as char induces changes in microbial communities and has structural features that favor microbial adhesion. Our results on the plant biomass and other physiological parameters (Table 1 and Table 2) were consistent with this idea. In addition, the molecular data on soil microbiome and gene expression indicated the same (Figure 1, Figure 2, Figure 3 and Figure 4). Moreover, to better analyze the effectiveness of the treatments, the three datasets were combined and ranked. The results obtained are reported in Table 3 and they showed that in wheat, the treatments with the largest contribution to the cultures were Char_MC-C, either with or without AMF, followed by Char_MC-B, either with or without AMF, and, similarly, for maize, the best growing conditions were for Char_MC-C, either with or without AMF, followed by Char_MC-B_AMF or AMF alone. This means that considering all data and species (plants and microbes), these treatments produced more positive results.

By considering the plant responses, physiological and gene expression data, and PCA analysis specific to wheat and maize, the results obtained confirmed that the treatments with Char_MC-C_AMF and Char_MC-B_AMF for wheat and maize, respectively, were those that accounted for most of the variances observed (Figures S6 and S7).

## 3. Materials and Methods

### 3.1. Strains and Growth Conditions

Two microbial consortia (MC-B and MC-C) were designed as part of the Horizon 2020 SIMBA project (Sustainable Innovation of Microbiome Applications in the Food System), as previously described [40]. Each of them combined five strains with different functional properties including PGPM; biocontrol strains; siderophore producers; strains producing alpha-amylase, alpha-glucosidase, and iso-amylase; and strains involved in N-fixation. MC-B was made up of *A. vinelandii* DSM 2289, *R. aquatilis* BB23/T4d, *Bacillus* sp. BV84, *B. amyloliquefaciens* LMG 9814, and *P. fluorescens* DR54. MC-C was composed of *A. chroococcum* LS132, *B. amyloliquefaciens* LMG 9814, *P. fluorescens* DR54, *B. ambifaria* MCI 7, and *R. aquatilis* BB23/T4d. The strains were kept at −80 °C in 30% glycerol for long-term storage.

Bacterial strains taken from cryopreserved pure cultures were streaked onto nutrient agar (NA) plates and grown at 28 °C for 24–48 h, then, the cells were transferred to an LB medium and incubated overnight in a thermostatic orbital shaker at 28 °C and 200 rpm, except for *A. vinelandii* DSM 2289, which was grown for 72 h due to its lower growth multiplication rate. Each culture was diluted at ratios of 1:2, 1:4, and 1:8, and the optical measurements (OD_600_) were assessed to provide the correlation of OD_600_ with the colony-forming units per mL (CFU mL^−1^) values that were obtained by serial dilution and plating of microbial suspension on NA plates.

The AMF (*R. intraradices*) was purchased from MycAgro lab, Bretenière, France (http://www.mycagrolab.com). The granular inoculum containing mineral solid particles (clay, zeolite), *R. intraradices* propagules (spores, hyphae pieces), and mycorrhizal root pieces (10 propagules/ gcontaining a mix of spores, mycelium, and mycorrhizal root pieces) [81] was added to soil at the concentration of 1.4% *w*/*w*.

### 3.2. Seed Coating and Liquid Delivery

Seeds of *T. durum* (cv Svevo) (kindly provided by Stuard farm, Parma, Italy) and of *Z. mays* (DKC6587) (Dekalb Monsanto Company, St. Louis, MO, USA), uncoated, were surface-sterilized using 10% sodium hypochlorite for 10 min and then subjected to three washes with deionized water. A microbial culture volume corresponding to 10^8^ CFU/ mlwas used for each strain. The consortium components were mixed at a 1:1:1:1:1 ratio and centrifuged and the pellet was resuspended in 500 μL of sterile 0.9% NaCl and transferred to 1% (*w*/*v*) sterile methyl cellulose. The seeds were incubated with this mix at 25 °C on a rotary shaker at 70 rpm for 1 h to allow the bacterial consortium to adhere to the seeds; then, the excess inoculum was removed, and the seeds were dried for 24 h before being sown. Control seeds were submerged in 50 mL of 1% methyl cellulose alone. For liquid delivery, each strain was mixed at a 1:1:1:1:1 ratio, each at a final concentration of 1 10^7^ CFU/mlin a sterile buffer (0.9% NaCl), and 5 mL were immediately supplemented to the plants.

### 3.3. Biochar Functionalization

Before being used as a carrier, biochar needs to be characterized [14]; thus, the necessary amount was weighed (0.1% *w*/*w* was used) and then sterilized. Fresh pre-cultures of each strain were prepared at a concentration of 10^7^ CFU/mLin LB and combined with the char (10 mL medium/ gof biochar). Cells were grown for 24 h at 28 °C with mild agitation; the medium was then drained and the char was rinsed with sterile water and immediately mixed with soil [15,82]. To assess functionalization, the biochar was observed using an AXIO Image Z2 (Zeiss, Jena, Germany) microscope and stained with Syto-9 (Thermo Fisher Scientific Inc., Waltham, MA, USA) at a final concentration of 1 μM. The microbial activity in the functionalized biochar was assessed by an XTT (3-Bis-(2-Methoxy-4-Nitro-5-Sulfophenyl)-2H-Tetrazolium-5-Carboxanilide) reduction assay as previously reported [83].

### 3.4. Experimental Setup in Greenhouse

Wheat and maize seeds were surface-sterilized using 10 % sodium hypochlorite for 1 h. Seeds were washed with sterilized dd H_2_O and sown in pots of 3 L using universal soil (Vigorplant Italia s.r.l., Fombio Lodi, Italy) mixed with natural sand. The soil composition was Baltic peat (21%), Irish peat (37%), volcanic pumice (13%), and superfine peat (29%); the physicochemical characteristics were pH (H_2_O) = 6.0–7.0 and EC = 0.30–0.40 dS /m. Plants were grown in a glasshouse at 25 °C during the day and 19 °C at night under supplemental lighting providing a minimum of 150 mmol m^−2^ /sphotosynthetic photon flux (14 hr day/10 hr night) for 60 days. Pots were disposed of in a fully randomized scheme and their positions were periodically swapped; they were watered daily with deionized water to maintain approximately 80% of the water-holding capacity of the soils. No further microbial or chemical fertilization was provided throughout the experiment.

In total, twelve and eight conditions were considered for wheat and maize, respectively, and six pots with three seeds per pot were prepared for each treatment. Specifically, for each treatment, two plants were used to sample leaf tissues at 21 and 60 DAS for transcriptomic analysis, whereas one plant per pot was used for physiological analyses.

At 15 DAS, for each pot containing an MC, a further liquid delivery was carried out with each consortium (MC-B or MC-C) as described above. Both the wheat and maize plants were harvested at 62 DAS.

### 3.5. Plant Growth Parameters

At 60 DAS, the chlorophyll content was measured in vivo with the SPAD-502 chlorophyll meter (Konica Minolta Business Solution Italia Spa, Milan, Italy), which was tested on three expanded leaves per plant. Ten measures were taken along the entire length of the leaf and the average was recorded. At 62 DAS, the plants were removed from the pots and washed with water and the excess water was dried out with absorbent paper. For each plant, the following growth parameters were measured on the shoots and roots: length, FW, and DW. Lengths were measured with a ruler (cm), whereas the weights were established with an analytical balance (g). Shoots and roots were oven-dried at 60 °C for 24 h to reach a constant dry weight and the dry weight data were recorded. The dry biomass ratio was calculated as the percentage of the dry weight with respect to the fresh weight for both roots and shoots. All data are presented as mean ± SD and were analyzed by ANOVA followed by Dunn’s post hoc test. A *p* value ≤ 0.05 was considered statistically significant. Data were analyzed using the software Past v. 3.14 [84].

### 3.6. Metabarcoding Analysis of Rhizosphere Soil

Rhizosphere soil was collected from each plant by shaking the roots, removing loosely adhered soil particles, and detaching the soil with a sterile chisel from different parts of the root system. Five to six grams of fresh rhizosphere soil were collected for each plant and stored at −80 °C until use. Genomic DNA was isolated from 250 mg of each sample of rhizosphere soil collected at 62 DAS using NucleoSpin^®^ Soil (Macherey-Nagel, Duren, Germany) according to the manufacturer’s instructions. From each sample, 50 ng of DNA was used to amplify the genes encoding 16S rRNA (V3-V4 region, primers: 16S f: 5′-TCGTCGGCAGCGTCAGATGTGTAAGAGACAGCCTACGGGNBGCASCAG-3´ and r: 5´-GTCTCGTGGGCTCGGAGATGTGTATAAGAGACAGGACTACNVGGGTATCTAATCC-3) as described in [85], and 18S (ITS2 region, 18S f: 5′-TCGTCGGCAGCGTCAGATGTGTATAAGAGACAGGCATCGATGAAGAACGCAGC-3′ and r: 5′-GTCTCGTGGGCTCGGAGATGTGTATAAGAGACAGTCCTCCGCTTATTGATATGC-3′) as reported in [86]. DNAs were amplified by PCR using the following procedures: 3 min initial denaturation at 98 °C, followed by 30 cycles with 30 s denaturation at 98 °C, 30 s annealing at 55 °C, 1 min elongation at 72 °C, and a 10 min final extension at 72 °C. Next-generation sequencing (NGS) was performed by BMR Genomics Srl (Padua, Italy) using standard procedures [87,88].

### 3.7. Target Genes Selection and Primers Design

Since the annotation for the maize genome is more advanced than for durum wheat, the target gene selection started with maize using the Maize database (www.maizegdb.org accessed on 12 April 2022, Assembly: Zm-B73-REFERENCE-NAM-5.0) and then the orthologous genes were identified for T. durum cv Svevo through a Blast search on the Ensembl database (plants.ensembl.org, Assembly: GCA_900231445.1). A list of the selected sequences is reported in Table S3. Primers were designed using Primer Express software v 2.00 (Applied Biosystems Inc., Foster City, CA, USA) and they are listed in Table S4. Each primer pair utilized in quantitative reverse transcription PCR (RT-qtPCR) was tested to assess its efficiency and specificity for the target genes of *T. durum* and *Z. mays*.

### 3.8. Expression Analysis by RT-qtPCR

Total RNA was isolated from 100 mg of leaf tissue using the RNeasy^®^ Plant Mini Kit (Qiagen GmbH, Hilden, Germany) according to the manufacturer’s instructions. The RNA concentration was determined using a spectrophotometer VARIAN Cary 50 UV-VIS (Agilent Technologies, USA). The total RNA (500 ng) was reverse-transcribed into cDNA using a Quantitect^®^R Reverse Transcription kit (Qiagen, Germany) according to the manufacturer’s instructions. The subsequent RT-qtPCR was based on 20 ng of a cDNA template and SsoAdvancedTM Universal SYBR^®^ Green Supermix 2X (Bio-Rad, Hercules, CA, USA) with 250 nM of each forward and reverse primers (Table S4). The reaction was conducted in a CFX96 Touch Real-Time PCR Detection System (Bio-Rad, USA) using the following procedures: 95 °C for 5 min, followed by 40 cycles at 95 °C for 15 s, 60 °C for 60 s, immediately followed by a melting curve analysis. The data were analyzed with the 2^−ΔΔCt^ method using 18S rRNA (18S) as a housekeeping gene and the control samples (without char or MC) as the calibrators [89]. The RT-qtPCR data are presented as the mean values calculated from three technical replicates and two biological replicates. The ΔCT values were visualized through a heatmap using the Heatmapper software (http://www.heatmapper.ca/ accessed on 12 June 2022). Ct values > 36 were considered undetermined. Contamination was excluded by the analysis of a negative control. Genes showing fold changes (FC) ≥ +2 or FC ≤ 0.5 were considered differentially expressed.

## 4. Conclusions

Biostimulants are natural products whose synthesis occurs without the inclusion of any contaminants. The biofertilizer market is projected to grow at a rate of 14.08% from 2016 driven by factors such as an increase in demand for fertilizers due to the rise in global food production and the development of new biofertilizer manufacturing technologies. Therefore, biostimulants promise to become a new class of biotechnological fertilizers capable of improving plant health and production but with more attention to sustainability and a reduction in the use of classic fertilizers but with significant environmental benefits (Figure S1). These benefits can span several crops from those for food production to those for feed and industrial purposes. To enlarge the performance of biostimulants, many delivery systems have been studied for cargo delivery with macromolecules such as alginate, hydroxyapatite, or materials such as organic matter or biochar [44]. Biochar produced from plant residues and agricultural wastes constitutes a good amendment that improves important soil properties. Moreover, biochar increases the CO_2_ sinking capacity of agricultural soils and can contribute to abating the presence of some contaminants [90].

The innovations in this paper as documented in the Results and Discussion section were (i) the testing of complex MCs with variances with a vast literature and single strains; (ii) the exploitation of char as a new delivery system for these MCs with a higher cargo capacity than previously reported systems; (iii) the reference to general crosstalk between soil and plants as a response to different treatments; (iv) a detailed bioinformatic and statistical analysis of all metagenomics and gene expression data with some system biology insights; and (v) the generation of an arbitrary evaluation matrix that includes all outputs with the purpose of generating a tool for researchers and practitioners for the possible use of MCs and char for more sustainable cropping of wheat and maize.

**Figure 5:**
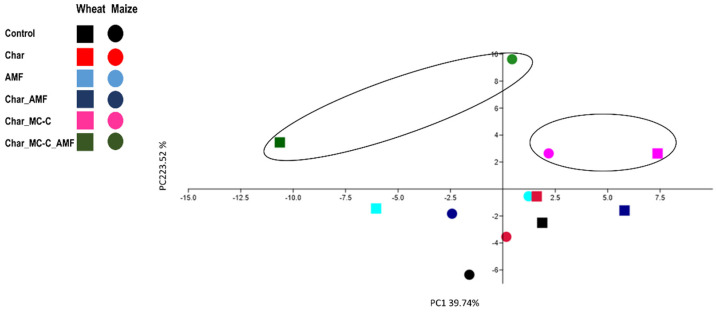
PCA analysis obtained with phenotypic and molecular (gene expression) data in the six growth conditions common to both wheat and maize plants (treatments and control). The black circles indicate the main grouping.

The evaluation matrix in Table 3 indicates a high ranking when supplements were used with char combined with MC (especially MC-C) and AMF for both crops. The PCA analysis conducted on the only “plant” data (physiological and gene expression) largely agrees with this and shows the major areas of the variances of these treatment conditions (Figure 5).

## Figures and Tables

**Table 1:** Measurements of the physiological parameters analyzed for wheat shoots and roots under the different treatments. For each trait, the mean value and standard deviation are reported.

Group	Length (cm)		Fresh Weight (FW) (g)		Dry Weight (DW) (g)		Dry Biomass %		Chlorophyll Content (SPAD)
Treatments	Roots	Shoots	Roots	Shoots	Roots	Shoots	Roots	Shoots	Leaves
***1_Control***									
**Control**	61.2 ± 6.7 ^b^	33.3 ± 2.6 ^b^	0.7 ± 0.1 ^b^	0.4 ± 0.1 ^b^	0.1 ± 0.1 ^b^	0.12 ± 0.02 ^b^	11.4± 3.4 ^bc^	28.1 ± 2.7 ^ab^	25.8 ± 2.2 ^abc^
**Char**	26.2 ± 2.3 ^c^	32.0 ± 5.1 ^b^	0.7 ± 0.3 ^b^	0.6 ± 0.1 ^b^	0.1 ± 0.0 ^b^	0.16 ± 0.03 ^b^	15.5± 6.1 ^bc^	28.1 ± 6.2 ^ab^	27.2 ± 2.3 ^abc^
**AMF**	64.6 ± 9.0 ^ab^	31.8 ± 4.3 ^b^	0.7 ± 0.1 ^b^	0.5 ± 0.1 ^b^	0.1 ± 0.0 ^b^	0.15 ± 0.04 ^b^	15.7± 3.2 ^bc^	26.7 ± 13.3 ^ab^	27.7 ± 1.6 ^ab^
**Char_AMF**	44.4 ± 3.4 ^c^	30.0 ± 3.7 ^b^	0.5 ± 0.1 ^b^	0.4 ± 0.1 ^b^	0.1 ± 0.0 ^b^	0.31 ± 0.03 ^b^	14.6± 3.2 ^bc^	**37.9 ± 10.8 ^a^**	24.1 ± 3.5 ^bc^
***2_Seed Coating***									
**MC-B**	53.6 ± 24.8 ^b^	35.4 ± 4.7 ^b^	0.7 ± 0.2 ^b^	0.5 ± 0.1 ^b^	0.1 ± 0.0 ^b^	0.15 ± 0.01 ^b^	12.5± 1.0 ^bc^	27.2 ± 4.3 ^ab^	23.6 ± 1.6 ^c^
**MC-C**	35.6 ± 17.2 ^bc^	27.8 ± 1.3 ^b^	0.6 ± 0.1 ^b^	0.4 ± 0.1 ^b^	0.1 ± 0.0 ^b^	0.14 ± 0.01 ^b^	11.4± 5.1 ^bc^	30.8 ± 7.6 ^ab^	24.2 ± 2.1 ^bc^
**MC-B_ AMF**	52.6 ± 8.1 ^ab^	28.4 ± 5.7 ^b^	0.4 ± 0.2 ^b^	0.4 ± 0.1 ^b^	0.1 ± 0.0 ^b^	0.15 ± 0.01 ^b^	18.5± 6.8 ^ab^	31.4 ± 4.6 ^ab^	22.2 ± 5.2 ^bc^
**MC-C_AMF**	39.4 ± 3.3 ^c^	31.6 ± 5.3 ^b^	**1.5 ± 0.5 ^a^**	0.6 ± 0.1 ^b^	0.1 ± 0.1 ^b^	0.16 ± 0.04 ^b^	9.9± 10.6 ^c^	27.2 ± 8.2 ^ab^	26.2 ± 3.3 ^abc^
***3_Functionalized Char***									
**Char_MC-B**	49.2 ± 9.4 ^b^	**45.0 ± 7.3 ^a^**	**1.6 ± 0.5 ^a^**	**1.9 ± 0.1 ^a^**	**0.2 ± 0.1 ^a^**	**0.47 ± 0.07 ^a^**	15.2± 7.8 ^bc^	24.1 ± 2.2 ^b^	**29.0 ± 4.1 ^a^**
**Char_MC-C**	48.6 ± 10.0 ^b^	**45.6 ± 3.3 ^a^**	**2.0 ± 0.7 ^a^**	**1.4 ± 0.4 ^a^**	**0.3 ± 0.1 ^a^**	**0.42 ± 0.02 ^a^**	14.7± 3.7 ^bc^	30.6 ± 8.4 ^b^	**33.3 ± 8.7 ^a^**
**Char_MC-B_AMF**	54.8 ± 3.3 ^b^	**45.4 ± 11.5 ^a^**	**1.6 ± 0.3 ^a^**	**1.7 ± 0.1 ^a^**	**0.3 ± 0.1 ^a^**	**0.44 ± 0.02 ^a^**	17.3± 3.0 ^ab^	28.4 ± 3.0 ^b^	**29.5 ± 1.5 ^a^**
**Char_MC-C_AMF**	**74.8 ± 7.0 ^a^**	**48.2 ± 3.3 ^a^**	0.6 ± 0.1 ^b^	**1.5 ± 0.3 ^a^**	0.2 ± 0.0 ^b^	**0.22 ± 0.06 ^a^**	**26.4 ± 6.7 ^a^**	16.0 ± 6.1 ^c^	27.8 ± 3.0 ^ab^

Different letters in the same column correspond to statistically different values (*p* < 0.05 one-way ANOVA, post hoc Dunn’s test). Values in bold are significantly different. Measurements were taken at 62 DAS.

**Table 2:** Measurements of the physiological parameters analyzed for maize shoots and roots when subjected to treatments as indicated. For each trait, the mean value and standard deviation are reported.

Treatment	Length (cm)		Fresh Weight (FW) (g)		Dry Weight (DW) (g)		Dry Biomass %		Chlorophyll Content (SPAD)
	Roots	Shoots	Roots	Shoots	Roots	Shoots	Roots	Shoots	Leaves
**Control**	51.7 ± 10.3 ^b^	56.0 ± 8.0 ^c^	3.1 ± 0.7	4.3 ± 1.7 ^c^	0.3 ± 0.1	0.6 ± 0.2 ^c^	10.3 ± 1.2	14.5 ± 1.4 ^ab^	9.4 ± 2.4 ^d^
**Char**	59.2 ± 16.6 ^b^	**68.0 ± 10.9 ^a^**	2.6 ± 0.5	5.0 ± 3.3 ^c^	0.3 ± 0.6	0.6 ± 0.3 ^c^	9.0 ± 1.0	12.0 ± 0.9 ^c^	13.0 ± 2.6 ^c^
**AMF**	**70.8 ± 9.3 ^a^**	62.8 ± 13.8 ^b^	3.1 ± 1.1	6.6 ± 2.9 ^abc^	0.2 ± 0.1	1.1 ± 0.7 ^abc^	8.7 ± 3.0	15.4 ± 2.7 ^ab^	12.2 ± 1.8 ^c^
**Char_AMF**	61.2 ± 10.5 ^b^	**68.3 ± 20.0 ^a^**	3.2 ± 1.7	5.3 ± 2.4 ^bc^	0.2 ± 0.1	0.7 ± 0.3 ^bc^	8.4 ± 1.9	13.0 ± 1.3 ^c^	13.4 ± 3.4 ^bc^
**Char_MC-B**	61.7 ± 13.0 ^b^	**64.5 ± 14.5 ^a^**	2.6 ± 0.4	4.8 ± 2.0 ^c^	0.2 ± 0.1	0.6 ± 0.3 ^c^	9.2 ± 0.7	14.3 ± 0.1 ^abc^	12.5 ± 2.3 ^c^
**Char_MC-B_AMF**	**88.2 ± 21.5 ^a^**	62.2 ± 9.6 ^b^	3.0 ± 0.9	**12.5 ±6.4 ^a^**	0.2 ± 0.1	**2.7 ± 0.1 ^a^**	8.1 ± 1.2	**22.3 ± 8.7 ^a^**	**17.5 ± 3.8 ^a^**
**Char_MC-C**	65.2 ± 7.0 ^b^	65.2 ± 9.1 ^abc^	3.0 ± 0.4	4.7 ± 1.8 ^c^	0.3 ± 0.1	0.7 ± 0.3 ^c^	9.1 ± 1.3	14.6 ± 2.2 ^ab^	15.0 ± 3.7 ^ab^
**Char_MC-C_AMF**	**81.0 ± 10.3 ^a^**	59.7 ± 11.8 ^b^	2.7 ± 1.0	8.9 ± 3.1 ^b^	0.3 ± 0.1	**1.5 ± 0.6 ^a^**	9.5 ± 1.1	16.1 ± 1.9 ^ab^	14.5 ± 3.4 ^ab^

Different letters in the same column correspond to statistically different values (*p* < 0.05 one-way ANOVA, post hoc Dunn’s test). Values in bold are significantly different. Measurements were performed at 62 DAS.

**Table 3:** Ranking the effectiveness of treatments in wheat and maize with a combination of physiological, metagenomic, and gene expression data. For each plant and treatment condition, the average values of the different parameters have been ranked and scores have been assigned ^$^. The scores have been summed over all parameters (column “Total”) and averaged (column “Mean”) to allow comparison of conditions with an unequal number of parameters. **Bold** indicates the treatment(s) with the highest ranking and *italics* indicate the treatment(s) with the lowest ranking.

**WHEAT**															
	Physiological Parameters *									**Metagenomic ^§^**		**Gene Expression ^#^**			
	**Length (cm)**		**FW (g)**		**DW (g)**				**Chlorophyll Content (SPAD)**	**Shannon Index**		**21d**	**60d**		
**Treatments**	**Root**	**Shoot**	**Root**	**Shoot**	**Root**	**Shoot**	**Root**	**Shoot**		**Bacteria**	**Fungi**	**Up**	**Up**	**Total**	**Mean**
Control	10	7	8	4	8	1	3	7	5	2	4	1	1	*61*	*4.7*
Char	1	6	8	8	8	7	8	7	7	1	1	7	7	76	5.8
AMF	11	5	8	6	8	5	9	3	8	6	4	3	2	78	6
Char_AMF	4	3	2	4	8	9	5	12	3	6	2	5	4	67	5.2
MC-B	8	8	8	6	8	5	4	5	2	n.t.	n.t.	n.t.	n.t.	54	6
MC-C	2	1	4	4	8	2	3	10	4	6	7	7	5	*63*	*4.8*
MC-B_AMF	7	2	1	4	8	5	11	11	1	n.t.	n.t.	n.t.	n.t.	50	5.6
MC-C_AMF	3	5	9	8	8	7	1	5	6	6	7	8	8	81	6.2
Char_MC-B	6	9	11	12	10	12	7	2	10	n.t.	n.t.	n.t.	n.t.	**79**	**8.8**
Char_MC-C	5	11	12	9	12	10	6	9	12	8	5	5	7	**111**	**8.5**
Char_MC-B_AMF	9	10	11	11	12	11	10	8	11	n.t.	n.t.	n.t.	n.t.	**93**	**10.3**
Char_MC-C_AMF	12	12	4	10	10	8	12	1	9	7	8	3	4	**100**	**7.7**
**MAIZE**															
	Physiological Parameters *									**Metagenomic ^§^**		**Gene Expression ^#^**			
	**Length (cm)**		**FW (g)**		**DW (g)**				**Chlorophyll Content (SPAD)**	**Shannon Index**		**21d**	**60d**		
**Treatments**	**Root**	**Shoot**	**Root**	**Shoot**	**Root**	**Shoot**	**Root**	**Shoot**		**Bacteria**	**Fungi**	**Up**	**Up**	**Total**	**Mean**
Control	1	1	7	1	8	3	8	4	1	5	8	1	2	50	*3.8*
Char	2	7	2	4	8	3	4	1	4	2	7	7	8	59	4.5
AMF	6	4	7	6	4	6	3	6	2	7	5	6	7	**69**	**5.3**
Char_AMF	3	8	8	5	4	5	2	2	5	2	6	6	2	58	4.5
Char_MC-B	4	5	2	3	4	3	6	3	3	6	4	6	5	54	4.2
Char_MC-C	5	6	5	2	8	5	5	5	7	8	2	8	4	**70**	**5.4**
Char_MC-B_AMF	8	3	5	8	4	8	1	8	8	5	3	2	4	**67**	**5.2**
Char_MC-C_AMF	7	2	3	7	8	7	7	7	6	5	1	6	7	**73**	**5.6**

n.t. = not tested. ^$^-In each column for each parameter, the greater values received the highest scores, corresponding to 12 or 8 according to the number of conditions tested. Other values received lower scores based on their position in the ranking, down to a minimum score of 1. * Physiological parameters from Table 1 and Table 2 have been ranked. ^§^ Metagenomic measures from Table S2 have been ranked. ^#^ For gene expression, we have considered the number of genes upregulated in each condition at 21 and 60 DAS; here, a gene was considered upregulated if its expression increased with a fold change ≥2.

## Data Availability

The data presented in this study are openly available at NCBI database, BioProject under accession number PRJNA875677 (https://www.ncbi.nlm.nih.gov/bioproject/PRJNA875677).

## Supplementary Materials

The supporting information can be downloaded at: https://www.mdpi.com/article/10.3390/ijms231810376/s1. References [42,89,91,92,93,94,95,96,97,98,99,100,101,102,103] are cited in the supplementary materials.
