# Supplementary material for: A Metagenomic and Gene Expression Analysis in Wheat (T. durum) and Maize (Z. mays) Biofertilized with PGPM and Biochar
Source: Int J Mol Sci. 2022 Sep 8;23(18):10376. doi: 10.3390/ijms231810376 (PMC9499264; doi:10.3390/ijms231810376)
Supplement: Supplementary file 1 [file ijms-23-10376-s001.zip › Supplementary material.pdf]

A metagenomic and gene expression analysis in wheat (*T. durum*) and maize (*Z. mays*) biofertilized with PGPM and Biochar

Sara Graziano [1], Marina Caldara [2], Mariolina Gullì [1,2], Annamaria Bevivino [3,] Elena Maestri [1,2], Nelson Marmiroli [1,2,4*]

[1] Interdepartmental Center SITEIA.PARMA, University of Parma, Parco Area delle Scienze, 43124 Parma, Italy;

[2] Department of Chemistry, Life Sciences and Environmental Sustainability, University of Parma, Parco Area delle Scienze 11/A, 43124 Parma, Italy;

[3] Department for Sustainability, Italian National Agency for New Technologies, Energy and Sustainable Economic Development, ENEA Casaccia Research Center, 00123 Rome, Italy;

[4]National Interuniversity Consortium for Environmental Sciences (CINSA), Venice-Parma, Italy

[*] Corresponding author:

E-mail address: nelson.marmiroli@unipr.it (N. Marmiroli).

Complete list of supplementary include:

Tables:

Table S1. Brief overview of main types of microbial delivery systems in plants.

Table S2. Shannon and Chao-1 index calculated for the bacterial and fungal populations of wheat and maize

Table S3. List of target genes of *T. durum* and *Z. mays* selected for transcriptional analysis (Excel file).

Table S4. Wheat and maize target gene sequences selection and primers design.

Figures:

Figure S1. Comparison in beneficial properties of the four main classes of fertilizers.

Figure S2. Functionalization of Char.

Figure S3. Rhizospheric bacterial communities at the level of class of wheat (A) and maize (B) soils untreated or treated with different combinations of biofertilizers.

Figure S4. Rhizospheric fungal communities at the level of class of wheat (A) and maize (B) soils untreated or treated with different combinations of biofertilizers.

Figure S5. Constructed network of maize selected genes by STRING software.

Figure S6. PCA analysis obtained with phenotypic and molecular (gene expression) data in the eight growth conditions of wheat plants (treatments and control).

Figure S7. PCA analysis obtained with phenotypic and molecular (gene expression) data in the eight growth conditions of the maize plants (treatments and control).

Table S1. Overview of main types of microbial delivery systems in plants.

Delivery system	Mode of action	References
Seed coating	Creating an envelope with a gelatinous or a polymer gel matrix that covers the seed and contains microbes and/ or other components (like micronutrients)	(Madden et al., 2012; Jambhulkar et al., 2016; Ma, 2019: Rocha et al. 2019)
Functionalized carrier (char, compost, clay, zeolite, perlite, talc, vermiculite)	Mixing liquid microbial cultures or lyophilized microbes with a solid dry material which will represent the major portion by volume and weight of the inoculant	(Daza et al., 2000; Malusá et al., 2012; Abd El-Fattah et al., 2013; Nuti & Giovannetti, 2015; Nehra and Choudhary, 2015; Singh et al., 2016; Duo et al., 2018)
Foliar application	Spraying liquid formulation directly on foliar parts (especially used in pathogens control)	(Esitken et al., 2006; Jambhulkar et al., 2016; Preininger et al., 2018; Abadi et al., 2021)
Microbigation	Appling the microbial consortia directly through the drip irrigation system	(Boari et al., 2008; Jambhulkar et al., 2016)

References (referred to Table S1)

Abadi, V.A.J.M., Sepehri, M., Rahmani, H.A., Zarei, M., Ronaghi, A., Taghavi, S.M., Shamshiripour, M., 2021. Correction to: Role of Dominant Phyllosphere Bacteria with Plant Growth–Promoting Characteristics on Growth and Nutrition of Maize (Zea mays L.). J. Soil Sci. Plant Nutr. 21, 2740–2740. https://doi.org/10.1007/s42729-021-00560-7

Abd El-Fattah, D.A., Eweda, W.E., Zayed, M.S., Hassanein, M.K., 2013. Effect of carrier materials, sterilization method, and storage temperature on survival and biological activities of Azotobacter chroococcum inoculant. Ann. Agric. Sci. 58, 111–118. https://doi.org/10.1016/j.aoas.2013.07.001

Boari, A., Zuccari, D., Vurro, M., 2008. "Microbigation": Delivery of biological control agents through drip irrigation systems. Irrig. Sci. https://doi.org/10.1007/s00271-007-0076-x

Daza, A., Santamaría, C., Rodríguez-Navarro, D.N., Camacho, M., Orive, R., Temprano, F., 2000. Perlite as a carrier for bacterial inoculants. Soil Biol. Biochem. https://doi.org/10.1016/S0038-0717(99)00185-6

Duo, L.A., Liu, C.X., Zhao, S.L., 2018. Alleviation of Drought Stress in Turfgrass by the Combined Application of Nano-compost and Microbes from Compost. Russ. J. Plant Physiol. https://doi.org/10.1134/S102144371803010X

Esitken, A., Pirlak, L., Turan, M., Sahin, F., 2006. Effects of floral and foliar application of plant growth promoting rhizobacteria (PGPR) on yield, growth and nutrition of sweet cherry. Sci. Hortic. (Amsterdam). 110, 324–327. https://doi.org/10.1016/j.scienta.2006.07.023

Jambhulkar, P.P., Sharma, P., Yadav, R., 2016. Delivery systems for introduction of microbial inoculants in the field, in: Microbial Inoculants in Sustainable Agricultural Productivity: Vol. 2: Functional Applications. pp. 199–218. https://doi.org/10.1007/978-81-322-2644-4_13

Livak, K., Piña, A., 2001. Analysis of Relative Gene Expression Data Using Real-Time Quantitative PCR and the 2−ΔΔCT Method Related papers Analysis of Relative Gene Expression Data Using Real-Time Quantitative PCR and the 2 CT M…. Methods 25, 402–408.

Ma, Y., 2019. Seed coating with beneficial microorganisms for precision agriculture. Biotechnol. Adv. https://doi.org/10.1016/j.biotechadv.2019.107423

Madden, T., Grentzer, J.M., Secura, G.M., Allsworth, J.E., Peipert, J.F., 2012. Risk of bacterial vaginosis in users of the intrauterine device: A longitudinal study. Sex. Transm. Dis. https://doi.org/10.1097/OLQ.0b013e31823e68fe

Malusá, E., Sas-Paszt, L., Ciesielska, J., 2012. Technologies for beneficial microorganisms inocula used as biofertilizers. Sci. World J. https://doi.org/10.1100/2012/491206

Nuti, M., Giovannetti, G., 2015. Borderline Products between Bio-fertilizers/ Bio-effectors and Plant Protectants: The Role of Microbial Consortia. J. Agric. Sci. Technol. A. https://doi.org/10.17265/2161-6256/2015.05.001

Nehra, V., Choudhary, M., 2015. A review on plant growth promoting rhizobacteria acting as bioinoculants and their biological approach towards the production of sustainable agriculture. J. Appl. Nat. Sci. https://doi.org/10.31018/jans.v7i1.642

Preininger, C., Sauer, U., Bejarano, A., Berninger, T., 2018. Concepts and applications of foliar spray for microbial inoculants. Appl. Microbiol. Biotechnol. https://doi.org/10.1007/s00253-018-9173-4

Singh, D.P., Singh, H.B., Prabha, R., 2016. Microbial inoculants in sustainable agricultural productivity: Vol. 2: Functional applications, Microbial Inoculants in Sustainable Agricultural Productivity: Vol. 2: Functional Applications. https://doi.org/10.1007/978-81-322-2644-4

Table S2. Shannon and Chao-1 index calculated for the bacterial and fungal populations of wheat and maize (Calculations were done on metagenomic data obtained at 60 DAS).

T. durum					*Z. mays*				
Bacteria	Shannon		Chao-1		Bacteria	Shannon		Chao-1	
	Mean	StDev	Mean	StDev		Mean	StDev	Mean	StDev
Control	4.38 abcd	0.07	202.5	7.8	Control	4.55	0.09	248.5	21.9
Char	4.28 a	0.15	200.1	16.3	Char	4.51	0.08	245.0	35.4
AMF	4.46 abcd	0.06	206.0	60.8	AMF	4.52	0.14	234.5	38.9
Char_AMF	4.46 abcd	0.06	231.5	51.6	Char_AMF	4.51	0.02	225.0	7.1
MC-C_AMF	4.53 b	0.13	219.0	25.5	Char_MC-B	4.60	0.03	241.0	17.0
MC-C	4.45 bc	0.00	192.7	45.3	Char_MC-C	4.66	0.08	243.0	9.9
Char_MC_C	4.54 bcd	0.15	241.0	59.4	Char_MC-B_AMF	4.57	0.04	238.5	29.0
Char_MC-C_AMF	4.52 abcd	0.07	253.5	4.9	Char_MC-C_AMF	4.54	0.11	244.0	1.4
Fungi	Shannon		Chao-1		Fungi	Shannon		Chao-1	
	Mean	StDev	Mean	StDev		Mean	StDev	Mean	StDev
Control	2.37 abc	0.00	35.5	6.4	Control	2.27	0.30	29.5	6.4
Char	2.15 a	0.12	36.5	9.2	Char	2.24	0.04	35.0	5.7
AMF	2.38 abc	0.59	48.0	8.5	AMF	2.35	0.03	30.0	7.1
Char_AMF	2.25 ac	0.31	38.5	0.7	Char_AMF	2.20	0.08	34.5	0.7
MC-C_AMF	2.57 abc	0.11	42.0	5.7	Char_MC-B	2.14	0.27	29.5	0.7
MC-C	2.56 abc	0.11	34.0	0.0	Char_MC-C	1.76	0.22	25.5	2.1
Char_MC_C	2.48 abc	0.18	40.5	7.8	Char_MC-B_AMF	1.84	0.01	27.0	2.8
Char_MC-C_AMF	2.88 b	0.07	46.0	0.0	Char_MC-C_AMF	1.69	0.20	34.0	5.7

Table S3. List of target genes of *T. durum* and *Z. mays* selected for transcriptional analysis (see Excel file). For each gene different information is reported: Gene ID, function, biological role, description, Gene Ontology (GO) and KEGG pathway.

Table S4. Wheat and maize target gene sequences selection and primers design.

Species	Gene ID	Gene	Primer Sequence 5'→ 3'	Amplicon length (bp)
Triticum durum	TRITD5Bv1G173280	**sm2**	Forward GGACATGCTTCCTCCTGGTT<br>Reverse CGTGTGCCCCAGGATGTTTA	94
Zea mays	Zm00001eb105580		Forward CATGATGCCAGTTCTCGTCG<br>Reverse CTGCCACAATCTCCTGCATC	203
Triticum durum	TRITD2Av1G000490	**P450**	Forward TGACTAGATGCAAAATTCTCTACAC<br>Reverse AGGTGAGGAAGAAGCGAACG	270
Zea mays	Zm00001eb402820		Forward ACAAGGTGCTGGTGTTCAAC<br>Reverse CGGCGTTGAAGGAGATGAAC	150
Triticum durum	TRITD7Av1G253180	**oy1**	Forward AAGGCGTTGGCTGCCCTAAA<br>Reverse TACGGAGCCGATGCCTCAAA	91
Zea mays	Zm00001eb407780		Forward ACGTTAGAGGCCCTTTCACC<br>Reverse CGTCCTCTCTCGTCCCTCTA	172
Triticum durum	TRITD3Av1G053480	**ga2ox6**	Forward ATGCTCCGGGTGAACCACTA<br>Reverse AGCAAGGAGATGATCTGCGG	98
Zea mays	Zm00001eb283860		Forward CTCATCAGTACGGCTCCAGT<br>Reverse CGAAGAAGATCACCGACACC	180
Triticum durum	TRITD3Bv1G186340	**psbp6**	Forward GCGACAACGTTGCCCGTAG<br>Reverse GTTGCCGGACAGTATGTTGG	201
Zea mays	Zm00001eb150500		Forward GGGTGATGCGTTACGTTGTT<br>Reverse GCCTAAAACAAGCAGTGCCT	150
Triticum durum	TRITD6Bv1G002390	**fad1**	Forward GGGACTTCGGGGACTCCAA<br>Reverse TCTTCACTTCTGAACCCGGC	132
Zea mays	Zm00001eb058080		Forward ACTAGCGAGCGATGAGAAAGA<br>Reverse TCAAGCAGGGTCAAAAGCAC	169
Triticum durum	TRITD6Av1G218420	**cer1**	Forward GTCGTACGAGAACCTCCACG<br>Reverse CTCCGTTGGCGTCGTATTGG	191
Zea mays	Zm00001eb191580		Forward TCGCTCAAAGTCAAAACCGG<br>Reverse AACTCACCACCACTACCCTG	180
Triticum durum	TRITD3Bv1G153520	**nmt1**	Forward TGTATCTTTCGGACGCGGAG<br>Reverse ATGGCCACCAACTTTTAGCC	71
Zea mays	Zm00001eb360800		Forward GATGTGAGATGTGCGTGGAC<br>Reverse CAAAGCAGTGACGGGAATGT	162
Triticum durum	TRITD1Av1G187530	**pgk**	Forward ATGGATTTGCAGGGTCGTC<br>Reverse CTCTGTGGGCTGTTCCGAAT	283
Zea mays	Zm00001eb291670		Froward CCACTGTGACCGTATGAAGC<br>Reverse AGGGTCGTCGTTGTTCAAAC	173
Triticum durum	TRITD5Bv1G082010	**pyrk**	Forward GCTGGATGGTAGTGATGCCA<br>Reverse ATGCCCTCCCAAATAGGCTC	158

Zea mays	Zm00001eb168480		Forward TACGCCGTCTTGATCTCCTC<br>Reverse TGCCGTCCAGCTGTACATAA	165
Triticum durum	TRITD7Bv1G093200	**Gpa**	Forward AGTAGTTGGACTCCGGTCCT<br>Reverse GTAGTCCGCACCCATTAGCA	73
Zea mays	Zm00001eb056340	**agpll1**	Forward TCTTCTTTCTCTCTGCCCGC<br>Reverse CGACAGTGATGCCAGATCGA	172
Triticum durum	TRITD2Av1G154670	**sdq2**	Forward AGCTGCGGATCTCACACTG<br>Reverse GGCGTATCTTATTGGCTGCTG	86
Zea mays	Zm00001eb297970		Forward GTGGATGACTGTGTTGGCAA<br>Reverse CCAGAACCAGATTGCAGCAC	159
Triticum durum	TRITD7Av1G259410	**pgd**	Forward ATGGTCACGAAAGGCATCGT<br>Reverse TTGGCGACGTAGTCAGCATT	74
Zea mays	Zm00001eb389520		Forward CCTGGGAAGGAAGCTCTCAA<br>Reverse AAGTACCCCGTGCTAGCTAC	180
Triticum durum	TRITD4Bv1G029580	**aceS3**	Forward TCGCGAGAAGAGGGTACAAC<br>Reverse ACAATGGTGAACAGCGCCTT	73
Zea mays	Zm00001eb246720		Forward AGCCTGGCATTTGTGATTGG<br>Reverse ACATATGCAACCTCAACGCG	180
Housekeeping		*Zea mays* **18S**	Forward GCCATCCCTCCGTAGTTAGC<br>Reverse TGCGGCCCAGAACATCTAAG	103
		Triticum durum **18S**	Forward GCCTAGTAAGCGCGAGTCAT<br>Reverse ATCCGAACACTTCACCGGAC	104

Beneficial properties	Chemical fertilizers	Organic fertilizers	Nano-fertilizers	Biostimulants or biofertilizers (without carriers)
Price				
Availability				
Production (scaling-up)				
Use of renewable resources				
Concentration of inorganic contaminants				
Concentration of organic contaminants				
Distribution				
Legislation framework				

Figure S1. Comparison of up/down (green/red) behavior regarding nine beneficial properties for the four main classes of fertilizers. The classification is based on authors' interpretation of the literature cited in the manuscript: red, bad performance – green, good performance – grey, intermediate performance – white, depending on composition.

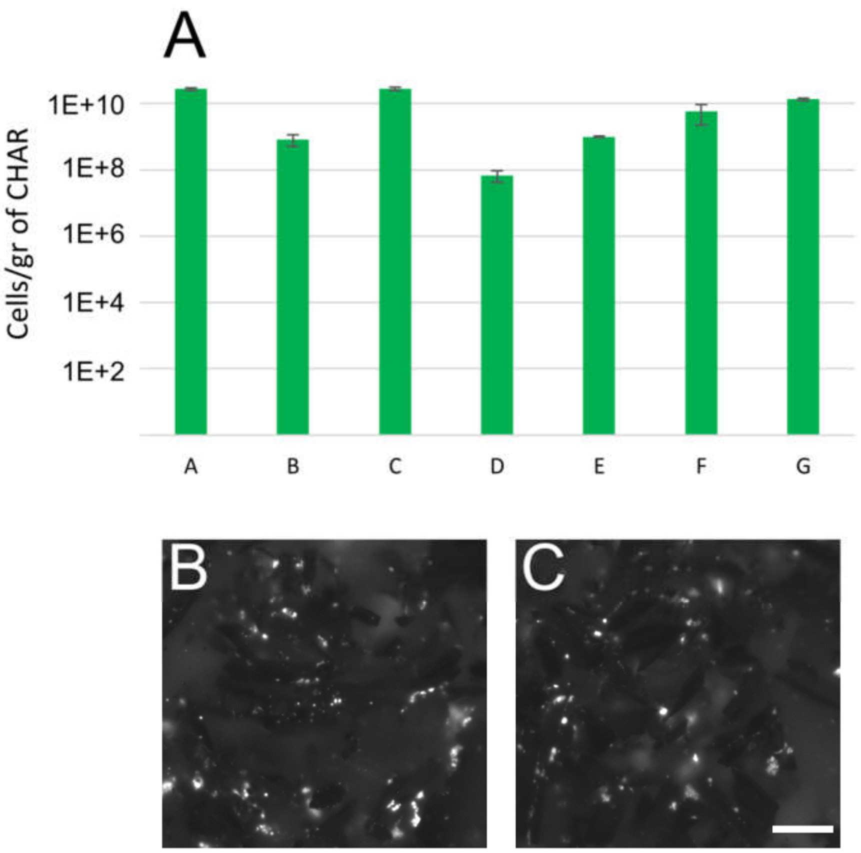

Figure S2. Functionalization of Char. (A) The y-axis is the Log cell counts per gram of biochar of each microbial consortium B and C after 24 hours of char functionalization in liquid medium. On the x-axis A: *R. aquatilis* BB23/T4d (member of MC-B and MC-C), B: *B. amyloliquefaciens* LMG 9814 (member of MC-B and MC-C), C: *P. fluorescens* DR54 (member of MC-B and MC-C), D: *A vinelandii* DSM 2289 (member of MC-B), E: *Bacillus sp*. BV84 (member of MC-B), F: *A. chrococcum* LS132b (member of MC-C) and H: *B. ambifaria* MCI 7 (member of MC-C). (B) and (C) staining of functionalized Char with Syto-9 of MC-B and MC-C, respectively. Scale bar is 10 µm.

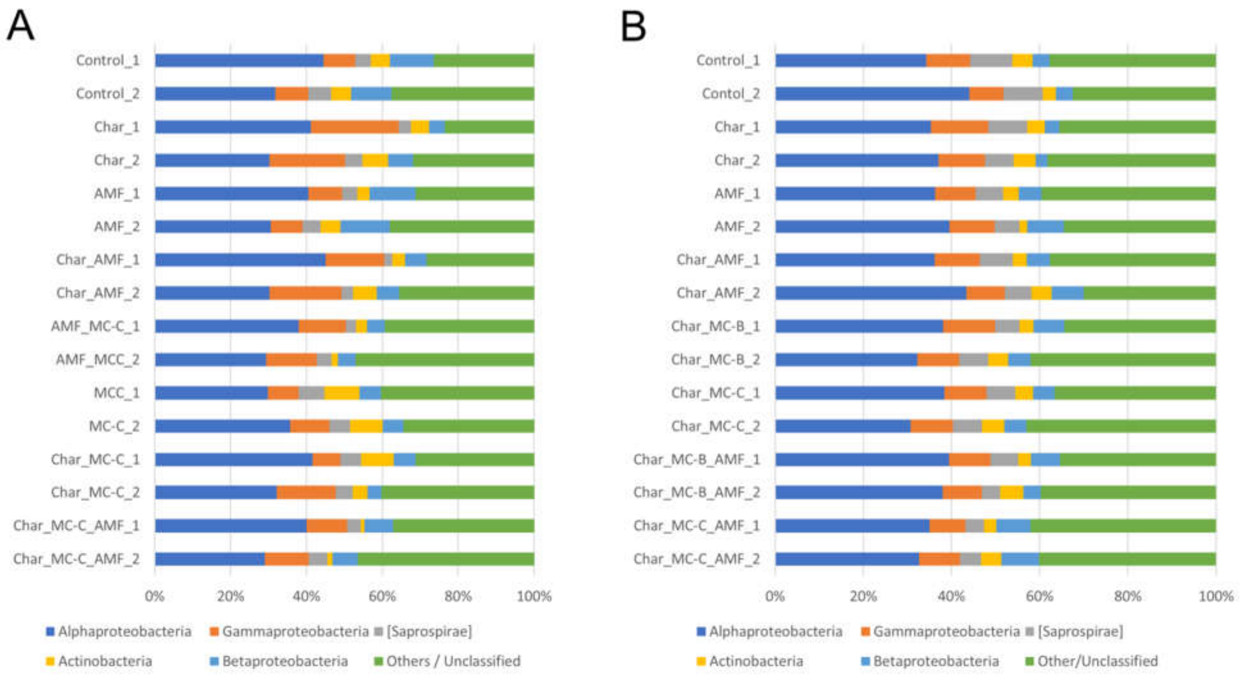

Figure S3. Rhizospheric bacterial communities at the level of class in wheat (A) and maize (B) soils untreated or treated with different combinations of biofertilizers. (A) and (B) report the relative abundance (based on OTUs) of the most abundant classes found in eight rhizospheric soil of wheat and maize analyzed in duplicate (1-2) the colors are ordered from left-to-right according to the legend reported at the bottom of the panel.

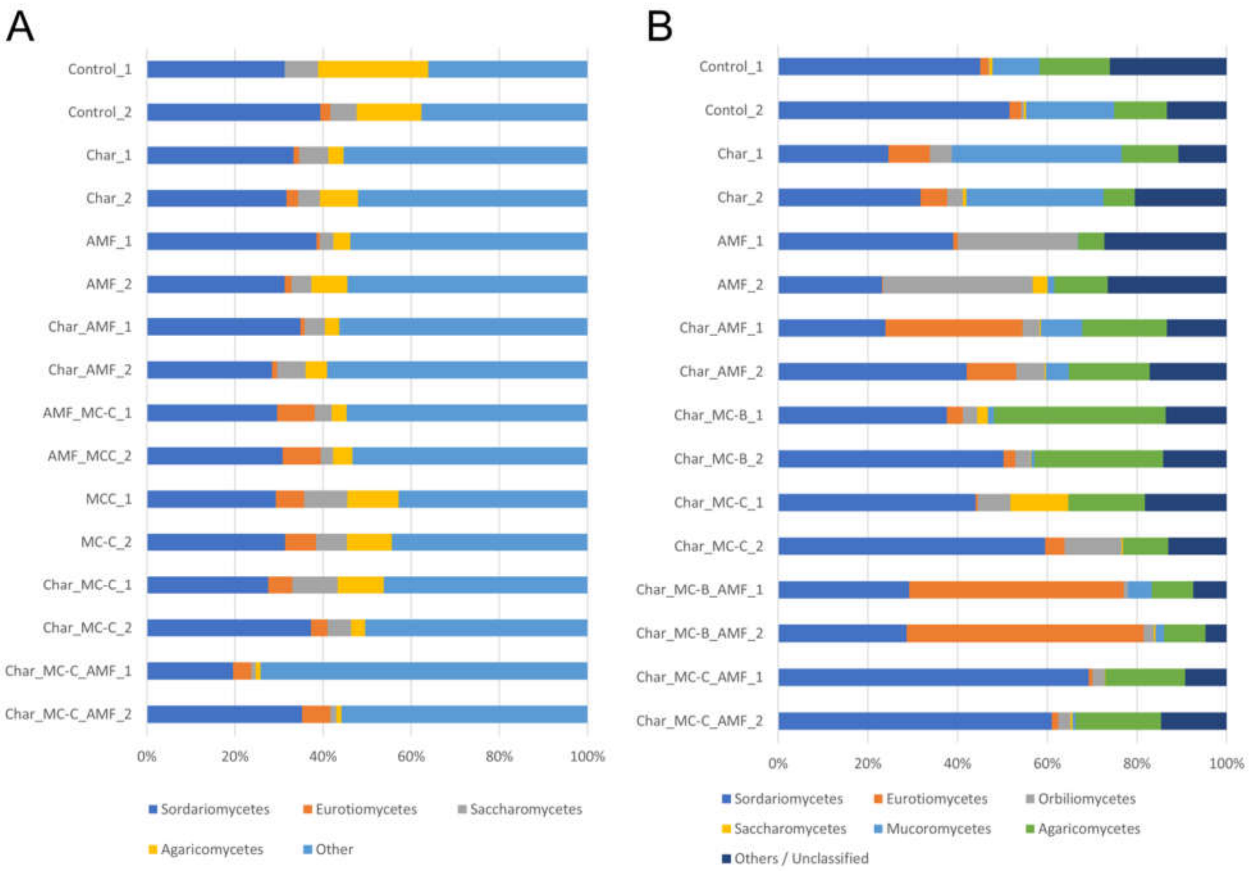

Figure S4. Rhizospheric fungal communities at the level of class in wheat (A) and maize (B) soils untreated or treated with different combinations of biofertilizers. (A) and (B) report the relative abundance (based on OTUs) of the most abundant classes found in eight rhizospheric soil of wheat and maize analyzed in duplicate (1-2) the colors are ordered from left-to-right according to the legend reported at the bottom of the panel

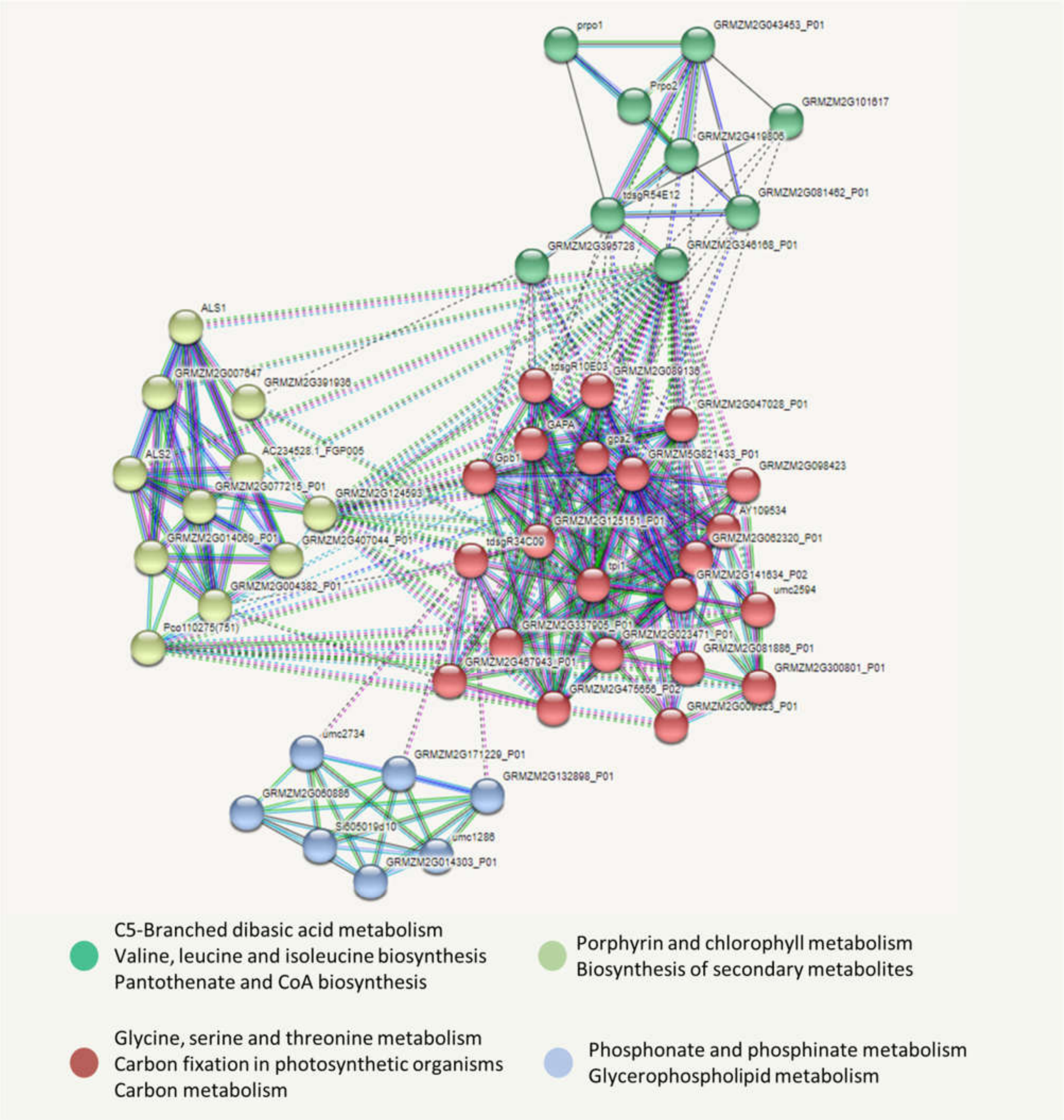

Figure S5. Network of the selected maize genes. Genes are represented with nodes and the interactions with continuous lines to represent physical direct interactions. Line thickness indicates the strength of data support. The colours present in the nodes of the network indicate the pathway in which each gene is involved. For analysis was utilized STRING software (https://string-db.org/) with k-means clustering methods with four numbers of clusters. Pathway analysis was performed by KEGG database.

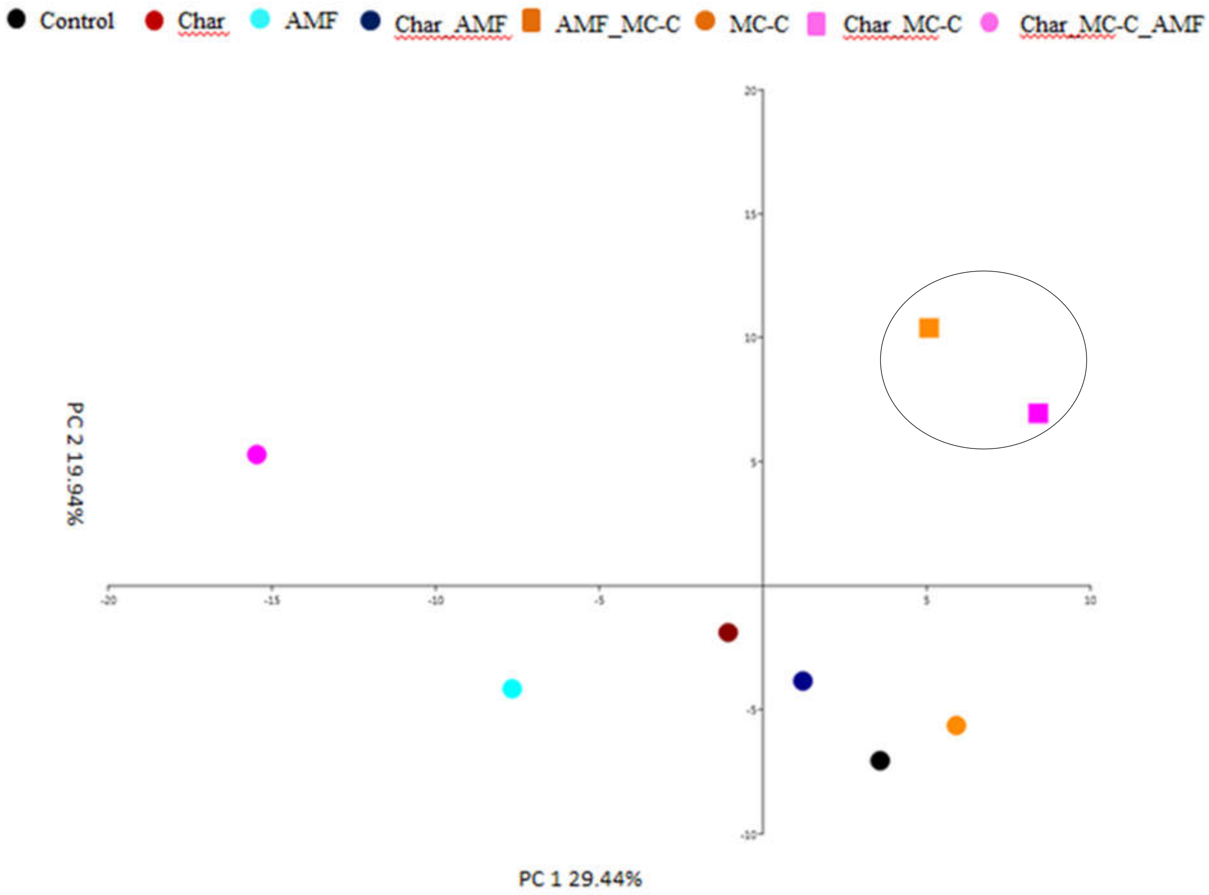

Figure S6. PCA analysis obtained with phenotypic and molecular (gene expression) data in the eight growth conditions of wheat plants (treatments and control). The main grouping is indicated by the black circle.

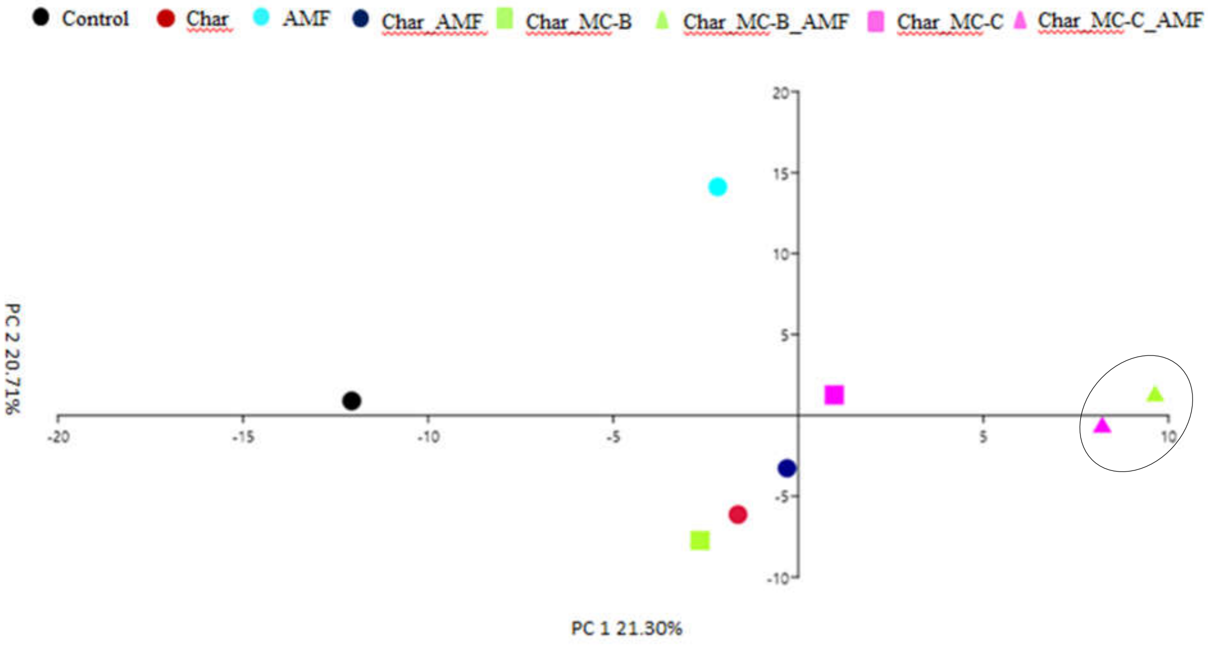

Figure S7. PCA analysis obtained with phenotypic and molecular (gene expression) data in the eight growth conditions of the maize plants (treatments and control). The main grouping is indicated by the black circle.
